# Elevated peripheral absolute monocyte count related to clinicopathological features and poor prognosis in solid tumors: Systematic review, meta‐analysis, and meta‐regression

**DOI:** 10.1002/cam4.3773

**Published:** 2021-02-16

**Authors:** Shu Wen, Nan Chen, Ying Hu, Litao Huang, Jin Peng, Meina Yang, Xiaoyang Shen, Yang Song, Liangzhi Xu

**Affiliations:** ^1^ Department of Obstetrics and Gynecology West China Second University Hospital Sichuan University Chengdu China; ^2^ West China School of Medicine Sichuan University Chengdu China; ^3^ Department of Thoracic Surgery West China Hospital Sichuan University Chengdu China; ^4^ Key Laboratory of Birth Defects and Related Diseases of Women and Children (Sichuan University Ministry of Education Chengdu China; ^5^ The Joint Laboratory for Reproductive Medicine of Sichuan University The Chinese University of Hong Kong Hong Kong China; ^6^ Department of Evidence‐Based Medicine and Clinical Epidemiology West China Hospital Sichuan University Chengdu China; ^7^ Department of Pharmacy Services Tacoma St. Joseph Medical Center CHI Franciscan Health System Tacoma WA USA

**Keywords:** inflammation, monocyte, prognosis, solid tumor

## Abstract

**Background:**

Absolute monocyte count (AMC) is often used to be assessed in cancer follow‐up, which has regained interest as a potential prognostic indicator in many solid tumors, though not consistently or comprehensively. In the present study, we set out to perform a comprehensive meta‐analysis of all available data regarding the prognostic significance of AMC in solid tumors. We also evaluated the association between AMC and clinical features in solid tumors.

**Methods:**

A hazard ratio (HR) and corresponding 95% confidence interval (*CI*) or a *p* value (*p*) from eligible studies were extracted and subsequently pooled analyzed. Subgroup analyses and meta‐regression analyses were conducted according to the confounders of included studies. In addition, the relationships between AMC and clinical characteristics were also explored in the meta‐analysis.

**Results:**

Overall, ninety‐three articles comprising 104 studies with 32229 patients were finally included. The results showed that elevated AMC was associated with worse overall survival (OS) *(HR *= *1*.*615*; *95% CI*: *1*.*475*‐*1*.*768*; *p* < *0*.*001)*, disease‐free survival (DFS) *(HR*:*1*.*488*; *95% CI*: *1*.*357*‐*1*.*633*; *p* < *0*.*001)*, progressive‐free survival (PFS) *(HR*: *1*.*533*; *95% CI*: *1*.*342*‐*1*.*751*; *p* < *0*.*001)* and cancer‐specific survival (CSS) *(HR*: *1*.*585*; *95% CI*: *1*.*253*‐*2*.*006*; *p* < *0*.*001)* in non‐hematological tumors. Subgroup analyses according to each confounder further proved the consistent prognostic value of AMC in solid tumor outcomes. Moreover, elevated AMC was more likely to be observed in male group and patients with smoking history, and associated with longer tumor length and advanced T stage.

**Conclusion:**

In short, the meta‐analysis found that elevated AMC might indicate poor long‐term outcomes in non‐hematologic cancers, thus AMC may be a valuable marker in the prognosis for patients with solid tumors.

## Graphical abstract

Elevated AMC indicates poor long‐term outcomes in solid tumors, which could be applied in the prognosis assessment.

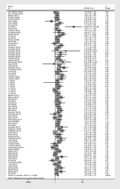

**Abbreviations**
AMC: absolute monocyte count
CCL2: C‐C chemokine ligand 2
CCR2: the receptor for chemokine CCL2
CI: confidence interval
CSS: cancer‐specific survival
DFS: disease‐free survival
HR: hazard ratio
MCP‐1: monocyte chemoattractant protein‐1
MDSC: myeloid‐derived suppressor cell
NK cell: natural killer cell
NOS: Newcastle–Ottawa Quality Assessment Scale
OR: odds ratio
OS: overall survival
PD‐L1: programmed cell death ligand 1
PFS: progressive‐free survival
PRISMA: Preferred Reporting Items for Systematic Reviews and Meta‐Analyses guidelines
RFS: recurrence‐free survival
ROC: receiver operating characteristic curve
SAR: survival after recurrence
SMD: standardized mean difference
TAM: tumor‐associated macrophage
TME: tumor microenvironment
VEGF: vascular endothelial growth factor

## 1 INTRODUCTION

Cancer remains one of the leading causes of death and a major health care challenge worldwide.^1^ The prominent differences of cancer profiles in individual countries are associated with marked geographic diversity.^1^ Despite substantial progress, cancer morbidity and mortality rates have been rapidly growing in both developing and developed countries.^1^ Based on the statistics, 1806,590 new cancer cases and 606,520 cancer deaths are projected to occur in the United States in 2020.^2^ Similarly, the Chinese national cancer registries reported about 3929,000 new cancer cases and 2338,000 cancer deaths in China in 2015.^3^

In the last decade, there have been the paradigms of chemotherapy being transformed from empiric therapy to individual pharmacogenomics‐ and genetics‐based personalized medicine, coinciding with rapid technological advances and brilliant discoveries in many fields such as genetics and molecular biomarkers.^4^ These markers have been recognized to reflect the characteristics of cancer signatures, optimize therapy decisions and provide timeliness information about the response to personalized treatment.^4^ While several biomarkers specific to particular cancer types have been applied routinely, such as PSA for prostate cancer, general decisive biomarkers used for overall cancers are unavailable.^5^ Thus, the identification of new potential tumor biomarkers with improved test convenience and sensitivity carries great significance to provide quicker diagnosis and more accurate prognosis.

With the increased understanding of tumor immunology, the dual interaction between cancer and the immune system has been recognized and the microenvironment in which the cancer cells grow has been highlighted.^6^, ^7^, ^8^ For a broad point of view, tumor microenvironment (TME) is a highly complicated heterogeneous ecosystem containing not only tumor cells, but also a variety of non‐immune and immune cells.^8^, ^9^ The immune contexture describes the density, function orientation, and spatial organization of the immune cell populations, including innate immune cells (e.g., macrophage, neutrophils, or natural killer cell (NK cell)), adaptive immune cells (e.g., T and B lymphocytes), and myeloid and lymphoid lineages.^9^ Immune cells play a pivotal role in the cytokine‐ and chemokine‐ mediated imbalanced immune response, which attributes to their inherent functions and the molecules they express.^10^ These cells have been reported to participate in the manifestation of tumor recognition and the consecutive steps of malignancy initiation, progression, and metastasis.^11^, ^12^ In addition, previous study indicated that tumor cells may modify the immunophenotype of immune cells and extracellular microenvironment, thus enhancing the deterioration of the immune contexture which determines tumor outcomes.^13^ The quantitative assessment of immunological status based on immune cells was applied in the prediction of some solid tumors.^14^

Tumor‐associated macrophages (TAMs), derived from the infiltrating bone marrow‐derived monocytes, are a major component in TME and therefore were considered as conspicuous stromal targets in many types of solid tumors.^6^, ^7^ Tumor‐associated macrophages could differentiate into “proinflammatory” M1 phenotypes with antitumor activity or “proangiogenic and immunosuppressive” M2 phenotypes according to the microenvironment. The dominant phenotype M2 was thought to be important in tumor progression, angiogenesis, and immune tolerance through promoting fibroblast proliferation, extracellular matrix deposition, and immunosuppression in the late stages.^15^, ^16^, ^17^ Recently, the rapid development of biotechnologies has boosted the understanding of the interplay between cancer cells and monocytes/macrophages.^18^ It is suggested that monocyte subpopulation distribution and transcriptomes are significantly perturbed by cancer, subsequently reflecting patients outcomes.^18^ In mouse models of cancer, monocytes have been shown to contribute to tumor progression, metastasis, and anti‐vascular endothelial growth factor therapy resistance.^19^, ^20^ Monocytes have been shown to be associated with cancer prognosis in patients with subdividing cancers, such as follicular lymphoma and colorectal cancer, although this line of research is still subject to debate.^21^, ^22^ Some data showed an insignificant prognostic value of monocytes in cervical cancer or malignant pleural mesothelioma.^23^, ^24^ Much more attention was paid to hematological tumors and lymphocyte‐to‐monocyte ratio.^25^, ^26^ Hence, the present researches available on the association between absolute monocyte count (AMC) and solid tumors have not been systematically analyzed so far, and evidence for the use of the peripheral AMC as a predictor of clinical outcome in solid tumors remained controversial.

Therefore, we performed a meta‐analysis with in order to validate the role of monocyte as a predictor in solid tumors. In addition, we also integrated data to demonstrate the relevant clinicopathological factors in relation to peripheral monocyte count, which is of the essence to tailor the personalized cancer treatment strategy.

## 2 METHODS

### 2.1 Data sources and search strategy

The present study was performed in accordance the Preferred Reporting Items for Systematic Reviews and Meta‐Analyses guidelines (PRISMA).^27^ An electronic literature search was conducted in the databases of Medline (PubMed), Embase, and the Web of Science on April 14, 2020, by two investigators independently, with the following search terms: “absolute monocyte count,” “monocyte count,” “cancer,” “carcinoma,” and “neoplasm.” In addition, manual searches were supplemented in all citation lists of the retrieved articles for further investigation of potentially relevant studies. Language was restricted to English and Chinese.

### 2.2 Criteria for inclusion and exclusion

Studies included in this meta‐analysis meet the following criteria: (1) patients with solid tumors were studied; (2) the prognostic impact of AMC on overall survival (OS), cancer‐specific survival (CSS), progressive‐free survival (PFS), disease‐free survival (DFS) and/or recurrence‐free survival (RFS) was evaluated; (3) a hazard ratio (HR) with 95% confidence interval (CI) could be extracted in univariate or multivariate analysis of Cox hazard model, or could be estimated by Parmar's method^28^; (4) AMC was calculated as a dichotomized variable by a cut‐off value. Studies were excluded if they match any of the following: (1) reference abstracts, case reports, conference abstracts, reviews or meta‐analysis; (2) studies on hematological malignancies; (3) insufficient data for estimating HR and 95% CI; (4) studies reporting AMC only as a continuous variable; (5) duplicate publications or repeated analysis. Moreover, if studies with overlapping patients were identified, the study with the most information and most recent publication was included. The full‐texts of the relevant articles were retrieved to assess eligibility.

### 2.3 Data extraction and quality assessment

Two investigators performed data extraction independently with a standard extraction form. The following data were extracted: first author's surname, publication year, country, region, patient sources, cancer type, study design, characteristics of cancer (distant metastasis, TNM stage, treatment), characteristics of the study cohort (sample size, mean age, gender), testing time of monocyte, cut‐off value defining low monocyte, method for the selection of cut‐off value, outcome measures (OS, DFS, CSS, RFS, PFS assessed as HRs and corresponding 95% CI and/or *p* values) model of survival analysis (multivariate or univariate).

The reviewers independently assessed the methodological quality of the included studies by the Newcastle–Ottawa Quality Assessment Scale (NOS).^29^ The 9‐point scoring system comprised three domains of quality including selection, comparability, and outcome assessment, and studies with NOS scores of more than six were defined as high‐quality studies. Any discrepancies between reviewers were resolved by discussion until a consensus was reached.

### 2.4 Statistical analysis

This meta‐analysis was performed with STATA version 14.0 (STATA Corporation, College Station, TX, USA). The survival data were measured by HR and 95% CI. The aggregated HRs and 95%CIs, either directly extracted or presented in the form of Kaplan‐Meier survival curves, were calculated to evaluate the prognostic value of AMC on the long‐term prognosis (OS/DFS/PFS/CSS) using low AMC group as a reference. The relationships between the AMC and certain clinical features of patients were also assessed with STATA version 14.0. Odds ratios (ORs) for dichotomous variables and standardized mean differences (SMDs) for continuous variables with their 95% CIs were regarded as summarized statistics, respectively. Statistical significance was indicated when *p* < *0*.*05* in data synthesis. Besides, we used the outcomes in multivariate analysis whenever the univariate and multivariate analyses were available. The heterogeneity of pooled studies was measured by Cochran's Q test and Higgins I‐squared *(*I^2^) statistic; *p* < *0*.*1* or *I* ^2^ > *50%* was defined as significant heterogeneity. The random‐effects model was used in the analysis. Subgroup analysis was performed based on region, sample size, cut‐off value, cancer type, distant metastatic status, TNM stage, testing time of blood, analysis method, and study quality to explore the heterogeneity sources. Then, meta‐regression analyses were conducted to determine the hazard effects of covariates. Publication bias was evaluated by Begg's and Egger's test. Sensitivity analysis was conducted to explore the influence on the pooled effect size after removing a single study each time. Two‐sided *p* < *0*.*05* was considered statistically significant.

## 3 RESULTS

### 3.1 Studies characteristics

**FIGURE 1:**
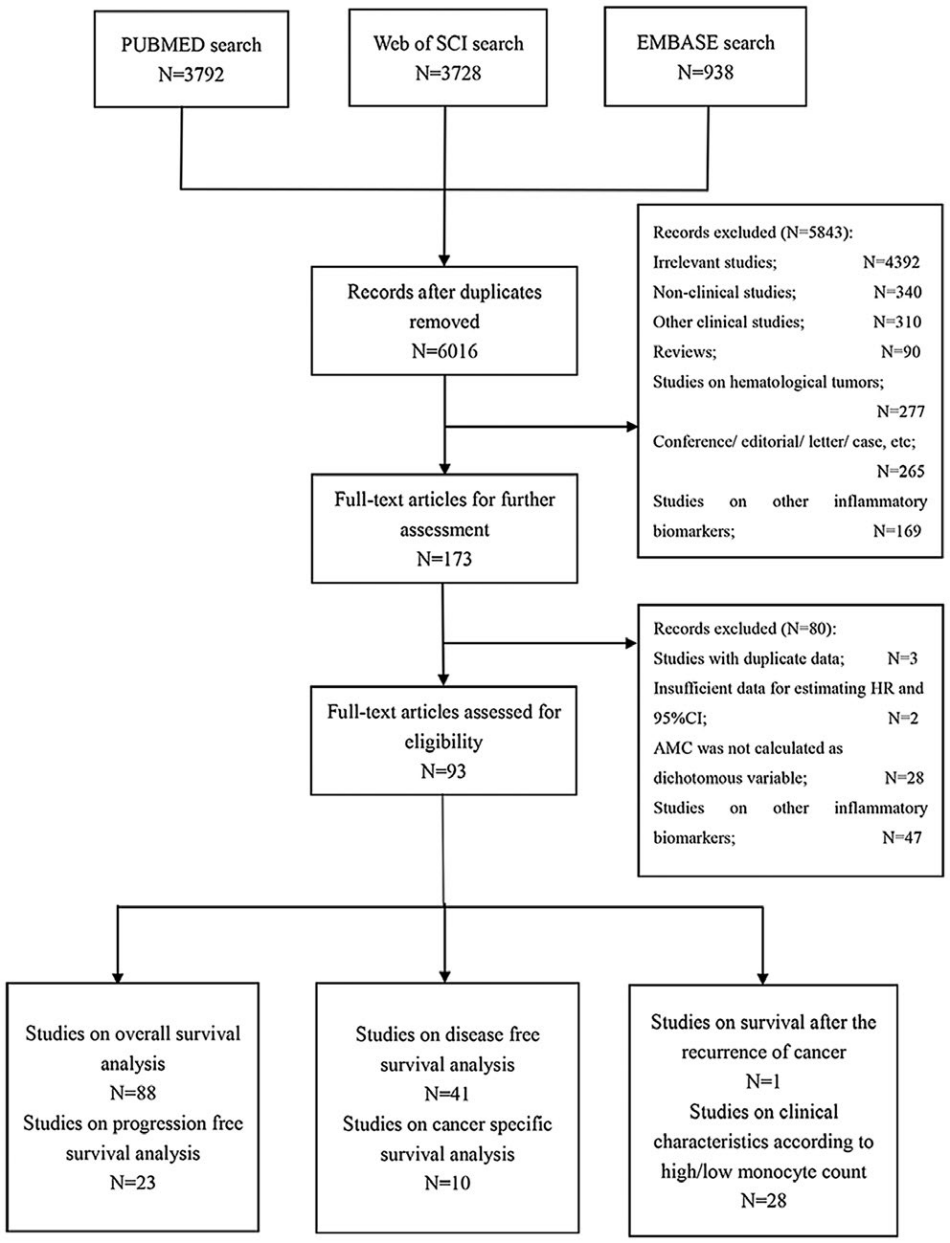
Flow chart of the literature search

The initial literature search identified a total of 6016 potentially relevant publications. After thoroughly screening the titles and abstracts by two investigators independently, the full‐texts of 173 potential studies were selected for further identification. Finally, 102 retrospective studies and 2 retrospective‐prospective studies from 93 eligible papers that met the inclusion criteria were included in this meta‐analysis.^22^, ^23^, ^24^, ^30^, ^31^, ^32^, ^33^, ^34^, ^35^, ^36^, ^37^, ^38^, ^39^, ^40^, ^41^, ^42^, ^43^, ^44^, ^45^, ^46^, ^47^, ^48^, ^49^, ^50^, ^51^, ^52^, ^53^, ^54^, ^55^, ^56^, ^57^, ^58^, ^59^, ^60^, ^61^, ^62^, ^63^, ^64^, ^65^, ^66^, ^67^, ^68^, ^69^, ^70^, ^71^, ^72^, ^73^, ^74^, ^75^, ^76^, ^77^, ^78^, ^79^, ^80^, ^81^, ^82^, ^83^, ^84^, ^85^, ^86^, ^87^, ^88^, ^89^, ^90^, ^91^, ^92^, ^93^, ^94^, ^95^, ^96^, ^97^, ^98^, ^99^, ^100^, ^101^, ^102^, ^103^, ^104^, ^105^, ^106^, ^107^, ^108^, ^109^, ^110^, ^111^, ^112^, ^113^, ^114^, ^115^, ^116^, ^117^, ^118^, ^119^ The flow diagram of the selection procedure is presented in Figure 1.

Of those 104 studies, 82 were based on Asians and 22 on non‐Asians. According to the types of cancer, 41 studies were on abdominal cancers, 30 studies on thoracic cancers, 14 studies on pelvic cancers, 12 studies on head and neck cancers, 6 studies on melanoma, and 1 study remained unknown. Based on the testing time of blood, majority of the included studies were pre‐treatment (*n* = 59) and pre‐operative (*n* = 42), while one study was post‐treatment and two studies remained unknown. There were 65 studies estimated as high‐quality studies and 39 low‐quality studies. The endpoints OS, DFS, CSS PFS, and survival after recurrence (SAR) were addressed in 88, 41, 23, 10, and 1 studies, respectively. As shown in Table 1, the receiver operating characteristic curves (ROC) were applied to detect the optimal cut‐off values in 40 included studies, 23 studies used median values and 4 studies used mean values, 12 studies chosen cut‐off values based on previous studies, 9 studies used cut‐offs with significant value of different statistical methods such as log‐rank test, 3 studies used a certain normal value, 3 studies used normal upper limits of monocyte count and 10 studies did not report methods determining the cut‐off values of AMC. The major characteristics of the meta‐analysis are shown in Table 1. The detailed extracted data are shown in Table S1 and detailed NOS scores of each included study are presented in Table S2.

**TABLE 1:** The baseline characteristics of included studies

Author, year	Region	Cancer type	Distant metastasis	TMN stage	Test time	N^a^	Mean age	Cut‐off value (/mm^3^)	Method^e^	Endpoint	Analysis	NOS score
Head and neck cancers												
Bobdey, 2017	Asian	Oral cavity cancer	Both	I‐IV	Pre‐treatment	471	50	500	ROC	OS	MV	8
Chen, 2009	Asian	HNC	Both	I‐IV	Pre‐treatment	270	56.5^b^	1000	Previous study	OS	MV	7
Furukawa, 2019	Asian	Tongue cancer	NA	I‐IV	Pre‐operative	103	63^b^	320	Mean value	OS	MV	7
Jiang, 2015	Asian	NPC	Yes	IV	Pre‐treatment	672	46	665	ROC	OS	MV	5
Li, 2013	Asian	NPC	No	I‐IV	Pre‐treatment	1547	51	475	ROC	OS/DFS	MV/MV	6
Lin, 2014	Asian	NPC	Yes	IV	Pre‐treatment	256	53.6	350	ROC	OS	MV	6
Huang, 2015^(1)^	Non‐Asian	OPC	NA	I‐IV	Pre‐treatment	510	57.5	600	Median value	OS/RFS	UV/UV	8
Huang, 2015^(2)^	Non‐Asian	OPC	NA	I‐IV	Pre‐treatment	192	65	700	Median value	OS/RFS	UV/UV	8
Takahashi, 2019	Asian	OPC	Both	I‐IV	Pre‐treatment	75	65^b^	485	ROC	OS/PFS	MV/MV	6
Tsia, 2014	Asian	Oral cavity cancer	Both	I‐IV	Pre‐treatment	202	53	403	Median value	CSS	MV	8
Yang, 2018	Asian	HPC	No	I‐IV	Pre‐treatment	197	NA	630	P value*	OS/DFS/CSS	UV/UV/UV	7
Yokato, 2020	Asian	Thyroid cancer	No	I‐IVA	Pre‐operative	570	58^b^	260	ROC	RFS	UV	6
Thoracic cancers												
Botta, 2013^(1)^	Asian	Lung cancer	Both	III‐IV	Pre‐treatment	73	58.57	600	ULN	PFS	UV	7
Botta, 2013^(2)^	Non‐Asian	Lung cancer	Both	III‐IV	Pre‐treatment	39	67.85	600	ULN	PFS	UV	7
Charrier, 2019	Non‐Asian	Lung cancer	Both	III‐IV	Pre‐treatment	148	62^b^	800	NA	OS/PFS	MV/MV	8
Chen, 2020	Asian	Breast cancer	No	II‐III	Pre‐treatment	262	48^b^	340	ROC	OS/DFS	UV/UV	7
Go, 2015	Asian	Lung cancer	Both	I‐IV	Pre‐treatment	134	68.5	640	Median value	OS	MV	6
Hai, 2018	Asian	Lung cancer	No	I‐IIIA	Pre‐operative	433	60.6	375	ROC	OS/DFS	UV/UV	9
Han, 2016	Asian	ESCC	No	I‐III	Pre‐operative	218	60.5	420	ROC	OS/DFS	MV/MV	9
Huang, 2015	Asian	ESCC	No	NA	Pre‐operative	348	59.2	550	ROC	CSS	MV	7
Huang, 2019(1)	Asian	Breast cancer	Both	I‐IV	Pre‐treatment	133	NA	440	NA	OS/DFS	UV/UV	7
Huang, 2019(2)	Asian	Breast cancer	Both	I‐IV	Pre‐treatment	317	NA	440	NA	OS/DFS	UV/UV	7
Huang, 2019(3)	Asian	Breast cancer	Both	I‐IV	Pre‐treatment	57	NA	440	NA	OS/DFS	UV/UV	7
Huang, 2019(4)	Asian	Breast cancer	No	I‐III	Pre‐treatment	94	NA	440	NA	OS/DFS	UV/UV	7
Kumagai, 2014	Asian	Lung cancer	No	I‐III	Pre‐operative	302	67	430	ROC	OS/RFS	MV/MV	8
Lee, 2017	Asian	Lung cancer	Both	III‐IV	Pre‐treatment	135	NA	800	Previous study	OS	MV	7
Lee, 2018(1)	Asian	Breast cancer	No	I‐III	Pre‐operative	NA	NA	360	Median value	DFS	UV	7
Lee, 2018(2)	Asian	Breast cancer	No	I‐III	Pre‐operative	37	NA	360	Median value	DFS	UV	7
Lee, 2018 (3)	Asian	Breast cancer	No	I‐III	Pre‐operative	NA	NA	360	Median value	DFS	UV	7
Lin, 2014	Asian	Lung cancer	Both	III‐IV	Pre‐treatment	370	63.6	450	ROC	OS/PFS	MV/MV	6
Ni, 2014	Asian	Breast cancer	No	I‐III	Pre‐treatment	542	49	400	Mean value	DFS	MV	7
Sakin, 2019	Asian	Lung cancer	Yes	IV	Pre‐treatment	113	65^b^	860	Normal value	OS	UV	6
Schernberg, 2017	Non‐Asian	ESCC	No	I‐III	Pre‐treatment	126	62	1000	NA	OS/PFS	UV/MV	7
Song, 2019	Asian	ESCC	No	I‐III	Pre‐operative	686	61^b^	500	Median value	OS/DFS	MV/UV	7
Soyano, 2018	Non‐Asian	Lung cancer	Both	NA	Pre‐treatment	157	66^b^	630	P value**	OS/PFS	MV/MV	7
Tang, 2016	Asian	Lung cancer	No	III	Pre‐treatment	78	57	600	Previous study	OS	UV	5
Tanizaki, 2018	Asian	Lung cancer	Both	III‐IV	Pre‐treatment	134	68	650	Previous study	OS/PFS	UV/UV	7
Tanrikulu, 2016	Non‐Asian	MPM	Both	I‐IV	Pre‐treatment	292	58.4	550	ROC	OS	MV	6
Wang, 2019	Asian	ESCC	Both	I‐IV	Pre‐treatment	43	62^b^	330	NA	OS	MV	6
Wen, 2015	Asian	Breast cancer	No	I‐III	Pre‐operative	2000	49.4	480	ROC	OS	MV	8
Zhang, 2017	Asian	MPM	NA	NA	Pre‐treatment	105	56	545	ROC	OS	MV	8
Zhu, 2017	Asian	ESCC	No	IIB	Pre‐operative	220	NA	630	Previous study	OS/DFS	UV/UV	9
Abdominal cancers												
Abu‐Shawer, 2019	Asian	Gastric cancer	Both	I‐IV	Pre‐treatment	502	54^b^	660	Median value	OS	UV	6
Cong, 2016	Asian	Gastric cancer	No	II‐III	Pre‐operative	188	77^b^	350	ROC	OS	MV	7
Feng, 2018	Asian	Gastric cancer	No	I‐III	Pre‐operative	3243	58^b^	510	X‐tile	OS	MV	6
Fujiwara, 2019	Asian	DEBDC	Both	I‐IV	Pre‐operative	121	66.7	300	ROC	OS/DFS	UV/UV	8
Giacomelli, 2017	Non‐Asian	Pancreatic cancer	No	I‐III	Pre‐treatment	57	62^b^	800	NA	PFS	UV	6
Gu, 2019	Asian	HCC	No	1‐III	Pre‐treatment	116	51	400	Median value	OS/RFS	UV/UV	7
Haruki, 2017	Asian	Colorectal cancer	Yes	IV	Pre‐operative	89	64	300	Mean value	OS/DFS	UV/UV	7
Hu, 2016	Asian	Colorectal cancer	Both	I‐IV	Pre‐operative	210	56.1^b^	505	ROC	OS	MV	7
Inamoto, 2019	Asian	Colorectal cancer	Both	I‐IV	Pre‐operative	448	69	400	ROC	OS/DFS/CSS	MV/MV/MV	6
Ishihara, 2019	Asian	Renal cell cancer	Yes	IV	Pre‐treatment	58	NA	650	Previous study	OS/PFS	UV/UV	7
Iwase, 2013	Asian	Gallbladder cancer	No	NA	Post‐operative	34	67	300	Previous study	OS/DFS	UV/UV	7
Kim, 2014	Asian	HCC	No	NA	Pre‐operative	256	54	300	ROC	RFS	UV	8
Krakowska, 2018	Non‐Asian	Colorectal cancer	Both	III‐IV	Pre‐treatment	295	63^b^	NA	Normal value	OS/PFS	UV/UV	7
Lee, 2016	Asian	Colorectal cancer	Yes	IV	Pre‐treatment	120	NA	413.3	ROC	OS	UV	6
Leith, 2007^(1)^	Non‐Asian	Colorectal cancer	No	I‐III	NA	149	NA	900	Previous study	OS/CSS	UV/UV	6
Leith, 2007^(2)^	Non‐Asian	Colorectal cancer	Yes	IV	NA	84	NA	900	Previous study	CSS	UV	6
Li, 2016	Asian	Pancreatic cancer	No	I‐III	Pre‐operative	144	62	400	ROC	OS/RFS	UV/UV	6
Li, 2018	Asian	Colon cancer	No	I‐III	Pre‐operative	216	64	350	Mean value	OS/PFS	MV/MV	7
Lin, 2014	Asian	HCC	Both	I‐IV	Pre‐treatment	216	64.8	380	ROC	OS	MV	9
Lin, 2016	Asian	Colorectal cancer	Yes	IV	Pre‐treatment	488	54	550	ROC	OS/PFS	MV/MV	6
Neal, 2015	Non‐Asian	Colorectal cancer	Yes	IV	Pre‐operative	302	64.8	700	ROC	OS/CSS	UV/UV	7
Oh, 2016	Asian	Colorectal cancer	No	II	Pre‐operative	261	65	520	ROC	OS/DFS	UV/UV	7
Paik, 2014	Asian	Colorectal cancer	Both	I‐IV	Pre‐operative	600	62.3	900	ROC	OS/DFS	MV/MV	6
Pan, 2018	Asian	Gastric cancer	No	I‐III	Pre‐operative	870	60^b^	230	ROC	OS	UV	7
Qi, 2015^(1)^	Asian	Pancreatic cancer	Both	III‐IV	Pre‐treatment	211	61.2	400	Median value	OS	MV	5
Qi, 2015^(2)^	Asian	Pancreatic cancer	Both	III‐IV	Pre‐treatment	110	60.8	400	Median value	OS	UV	5
Ren, 2016	Asian	HCC	No	NA	Pre‐operative	101	49.2	456	ROC	OS/DFS	UV/UV	8
Saito, 2019	Asian	Gastric cancer	No	I‐III	Pre‐operative	445	NA	658.5	ROC	OS	UV	6
Sasaki, 2006	Asian	HCC	NA	NA	Pre‐operative	198	63	300	Median value	DFS/CSS	MV/MV	9
Sasaki, 2007	Asian	Colorectal cancer	Yes	IV	Pre‐operative	97	62.6	300	Median value	CSS	MV	9
Shen, 2014	Asian	HCC	No	NA	Pre‐operative	351	50.1	545	ROC	OS/DFS	MV/MV	8
Shibutani, 2017	Asian	Colorectal cancer	No	I‐III	Pre‐operative	189	68	300	Previous studies	OS/RFS	UV/UV	6
Tanio, 2019	Asian	Colorectal cancer	Both	I‐IV	Pre‐operative	361	NA	421.5	ROC	OS	UV	7
Urakawa, 2019	Asian	Gastric cancer	No	II‐III	Pre‐operative	278	68^b^	401	Median value	OS/RFS	MV/MV	8
Wang, 2018	Asian	Gastric cancer	No	I‐III	Pre‐treatment	104	60	330	Median value	OS	UV	8
Wang, 2019	Asian	Gastric cancer	No	III	Pre‐operative	182	55.7	440	Median value	OS/DFS	UV/UV	7
Wu, 2019	Asian	Colorectal cancer	No	I‐III	Pre‐treatment	153	56^b^	330	Median value	OS	UV	8
Yamamoto, 2020	Asian	Colorectal cancer	No	I‐III	Pre‐operative	463	NA	455.5	ROC	OS	UV	6
Yang, 2017	Asian	Colon cancer	Yes	IV	Pre‐treatment	95	56	370	Median value	OS/PFS	MV/MV	7
Zhang, 2015	Asian	Rectal cancer	No	IIB	Pre‐operative	270	NA	595	ROC	OS/DFS	MV/MV	8
Zhang, 2016	Asian	ICC	Both	NA	Pre‐treatment	187	60^b^	500	Median value	OS	MV	6
Pelvic cancers												
Abu‐Shawer, 2019	Asian	GNC	Both	III‐IV	Pre‐treatment	259	57	590	ROC	OS/EFS	UV	6
Burgess, 2020	Non‐Asian	Endometrial cancer	Both	I‐IV	Pre‐operative	310	63.8	700^e^	Previous study	OS/ DFS/ PFS	MV/MV/UV	7
Eo, 2018	Asian	Cervical cancer	No	I‐II	Pre‐treatment	233	51^b^	800	Previous study	OS/PFS	MV/MV	8
Hayashi, 2017	Asian	Prostate cancer	No	I‐III	Pre‐operative	248	66	369	Median value	RFS	MV	6
Ittiamornlert, 2019	Asian	Cervical cancer	Both	I‐IV	Pre‐treatment	355	52.5	970	NA	OS/PFS	UV/UV	6
Lee, 2012	Asian	Cervical cancer	No	I‐IVA	Pre‐treatment	788	51	349	Median value	OS/PFS	MV/MV	6
Lee, 2020	Asian	Cervical cancer	No	II‐III	Pre‐treatment	125	53.67	330	ROC	OS/DFS	UV/UV	6
Li, 2016	Asian	Cervical cancer	No	II‐IVA	Pre‐treatment	424	47	380	ROC	OS/PFS	MV/MV	6
Machida, 2017	Non‐Asian	Endometrial cancer	Both	I‐IV	Pre‐treatment	141	58.2	400	NA	SAR	UV	8
Matsuo, 2015	Non‐Asian	Endometrial cancer	NA	I‐IV	Pre‐operative	541	52.1	700	P value***	OS/DFS	MV/MV	8
Shigeta, 2016^(1)^	Asian	Prostate cancer	Yes	IV	Pre‐treatment	106	73	400	ROC	OS/PFS	MV/MV	6
Shigeta, 2016^(2)^	Asian	Prostate cancer	Yes	IV	Pre‐treatment	108	71	400	ROC	OS/PFS	MV/MV	6
Singh, 2017	Non‐Asian	Cervical cancer	No	I‐IVA	Pre‐treatment	181	52	660	Median value	OS/PFS	UV/UV	8
Wang, 2017	Asian	Prostate cancer	Both	I‐IV	Pre‐treatment	290	75	425	ROC	OS/PFS/CSS	MV/UV/MV	8
Other cancers												
Gandini, 2016	Non‐Asian	Melanoma	Yes	IV	Pre‐treatment	127	55	460	ULN	OS	MV	7
Martens, 2016	Non‐Asian	Melanoma	Yes	IV	Pre‐treatment	209^c^	58	413.3	P value*	OS	UV	6
Rochet, 2015^(1)^	Non‐Asian	Melanoma	No	III	Pre‐operative	153	59^b^	600	P value****	OS/RFS	MV/MV	6
Rochet, 2015^(2)^	Non‐Asian	Melanoma	Yes	IV	Pre‐operative	74	56^b^	600	P value****	OS/RFS	MV/MV	6
Schmidt, 2005	Non‐Asian	Melanoma	Yes	IV	Pre‐treatment	321	51	NA	Normal value	OS	UV	5
Shi, 2020	Asian	Malignant cancers^d^	Both	NA	Pre‐treatment	193	65^b^	370	P value***	OS	MV	7
Wagner, 2020	Non‐Asian	Melanoma	No	I‐II	Pre‐operative	1412^c^	63	810	P value***	OS	MV	7

NOS: Newcastle‐Ottawa Quality Assessment Scale; OS: overall survival; DFS: disease‐free survival; PFS: progression‐free survival; SAR: survival after the recurrence of cancer; MV: multivariate analysis; UV: univariate analysis; ULN: upper limit of normal; DEBDC: distal extrahepatic bile duct cancer; ESCC: esophageal squamous cell carcinoma; GNC: Gynecological cancers; HCC: hepatocellular carcinoma; HPC: hypopharyngeal squamous cell carcinoma; ICC: intrahepatic cholangiocarcinoma; MPE: malignant pleural effusion; MPM: malignant pleural mesothelioma; NPC: nasopharyngeal carcinoma; OPC: oropharyngeal cancer; TET: thymic epithelial tumor; ROC: receiver operating characteristic curve; NA: not available information.

^a^ Number of included patients.

^b^ Median.

^c^ Existing missing data.

^d^ Malignant cancers with pleural effusion.

^e^ Method of determining cut‐off value.

* *p* value: the lowest significant log‐rank *p*‐value of all analyzed eccentric cut‐off points.

** *p* value: the cut‐off value was assessed by Contal and O'Quigley.

*** *p* value: the cut‐off value to maximize the survival outcome for DFS and OS.

**** *p* value: the significant value of *χ* ^2^ test of the log‐rank test analysis for different cut‐off points between the 25% and 75% quartile values.

### 3.2 Overall survival

**FIGURE 2:**
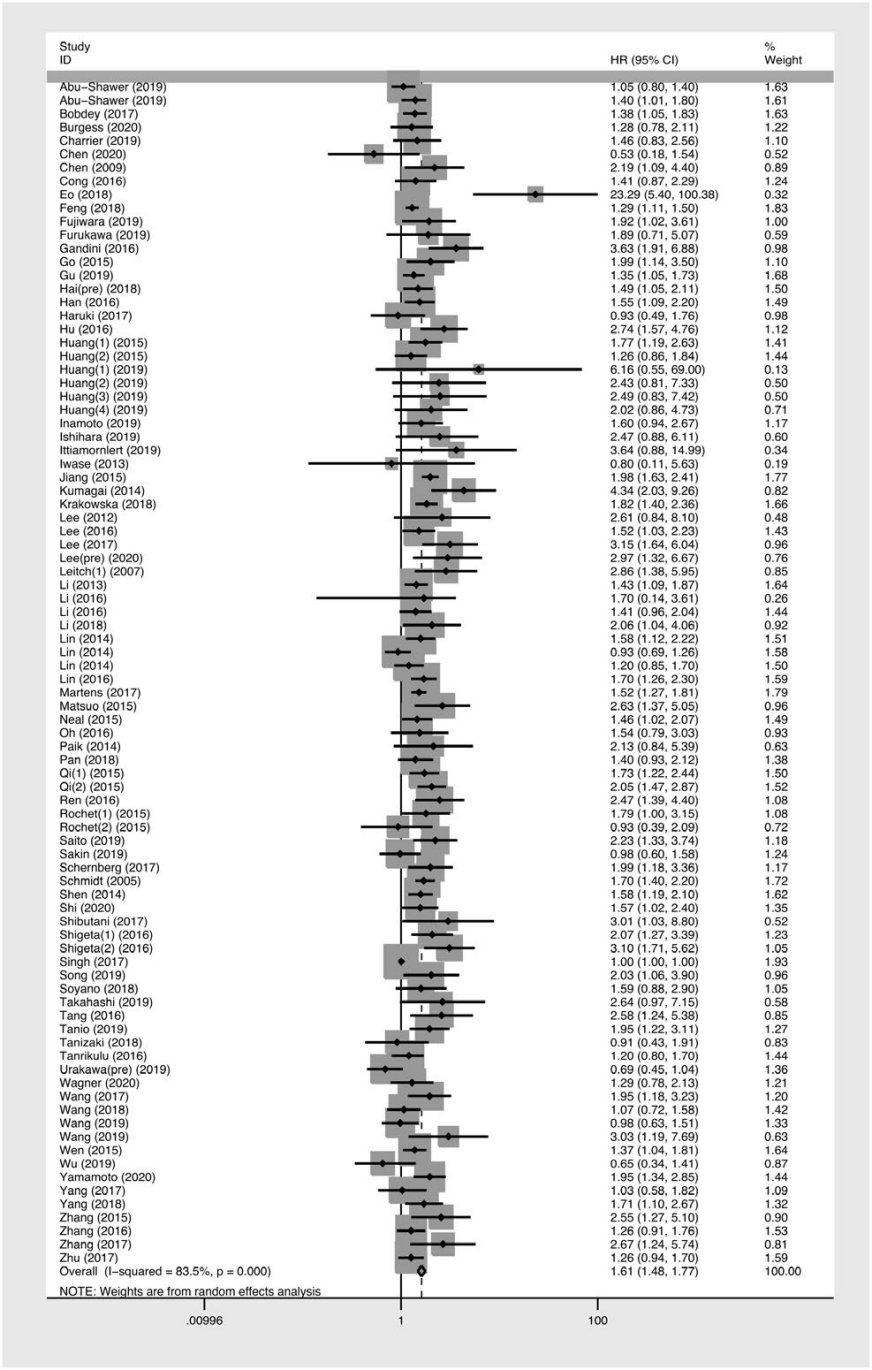
Forest plot of meta‐analysis of the prognostic role of absolute monocyte count for overall survival with random‐effects model

**FIGURE 3:**
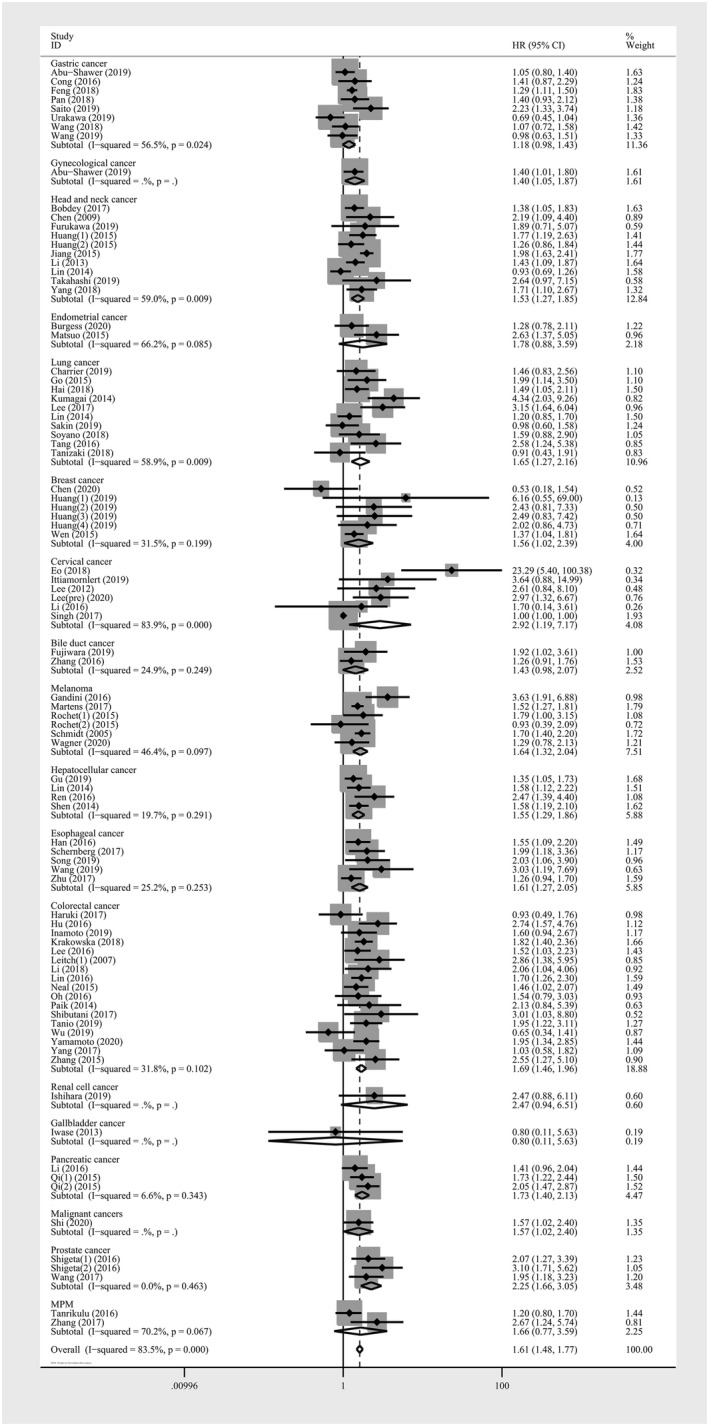
Forest plot of meta‐analysis of the prognostic role of absolute monocyte count for subgroup analysis of overall survival stratified by cancer type in solid tumors

A total of 88 studies including 29,130 patients provided suitable data for OS analysis. Comparing with low monocyte count, the elevated AMC showed a significant relevance with poorer survival *(HR *= *1*.*615*; *95% CI*: *1*.*475*‐*1*.*768*; *p* < *0*.*001)* (Figure 2). The test of heterogeneity was significant and random‐effects model was used *(I^2^ *= *83*.*50%*; *p* < *0*.*001)*. Subgroup analyses were performed based on the available data. The subgroup analyses revealed a significant association between higher AMC and unfavorable cancer prognosis in Asian patients, studies with large sample size, studies with low cut‐off value, studies with multivariate analysis, and low‐quality studies with decreased heterogeneity. Similar associations were seen in analyses stratified by TNM stage and distant metastatic status, as well as in pre‐treatment studies and pre‐operative studies. Considering various cancer types may lead to inconsistent results, a subgroup analysis according to cancer type was conducted. Most subgroups showed a negative prognostic effect of elevated AMC. This includes the subgroups of breast cancer, cervical cancer, colorectal cancer, esophageal cancer, hepatocellular cancer, head and neck cancer, lung cancer, melanoma, pancreatic cancer, and prostate cancer. Of subgroups stratified by cancer type, the highest effect on OS was found in cervical cancer *(HR*: *2*.*917*; *95% CI*: *1*.*186*‐*7*.*175*; *p* = *0*.*020)* and no heterogeneity was found in the prostate cancer subgroup *(HR*: *2*.*253*; *95% CI*: *1*.*665*‐*3*.*048*; *p* < *0*.*001)* (Figure 3). When stratified by primary tumor sites, the pooled highest effect on OS was found in pelvic cancers *(HR*:*2*.*111*, *95% CI*: *1*.*480*‐*3*.*011*, *p* < *0*.*001)*. (Table 2). Meta‐regression analyses were performed, while none of an individual parameter was identified as the cause of heterogeneity (Table 2).

**TABLE 2:** The pooled data on the survival of the meta‐analysis

Variables	N^a^	Case^b^	Pooled data		Heterogeneity		Meta‐regression (*p* value)
			HR (95% CI)	P	*I^2^*	*Ph*	
(High level vs. low level)							
*Overall survival*							
Overall	88	29,130	1.615 (1.475‐1.768)	<0.001	83.50%	<0.001	
By region							
Asian	70	23,730	1.589 (1.462‐1.728)	<0.001	53.90%	<0.001	0.791
Non‐Asian	18	5400	1.580 (1.307‐1.910)	<0.001	86.40%	<0.001	N. E.
By sample size							
>200	46	23,899	1.560 (1.431‐1.700)	<0.001	52.30%	0.001	0.886
≤200	42	5231	1.640 (1.419‐1.895)	<0.001	80.20%	<0.001	N. E.
By cut‐off value							
>500	37	13,786	1.657 (1.440‐1.907)	<0.001	84.50%	<0.001	0.664
≤500	48	14,728	1.543 (1.401‐1.700)	<0.001	49.50%	<0.001	0.543
NA	2	616	1.750 (1.475‐2.077)	<0.001	0%	0.699	N. E.
By cancer type							
Bile duct cancer	2	308	1.427 (0.984‐2.070)	0.061	24.90%	0.249	0.428
Breast cancer	6	2863	1.564 (1.023‐2.391)	0.039	31.50%	0.199	0.464
Cervical cancer	6	2106	2.917 (1.186‐7.175)	0.020	83.90%	<0.001	0.571
Colorectal cancer	17	4709	1.689 (1.456‐1.958)	<0.001	31.80%	0.102	0.533
Endometrial cancer	2	851	1.777 (0.880‐3.588)	0.109	66.20%	0.085	0.598
Esophageal cancer	5	1293	1.614 (1.268‐2.053)	<0.001	25.20%	0.253	0.543
Gallbladder cancer	1	34	0.800 (0.114‐5.632)	0.823			0.376
Gastric cancer	8	5812	1.181 (0.978‐1.426)	0.084	56.50%	0.024	0.233
Gynecological cancer	1	259	1.400 (1.049‐1.869)	0.022			0.404
Hepatocellular cancer	4	784	1.551 (1.292‐1.861)	<0.001	19.70%	0.291	0.496
Head and neck cancer	9	4293	1.530 (1.265‐1.850)	<0.001	59.00%	0.009	0.439
Lung cancer	10	2004	1.654 (1.265‐2.162)	<0.001	58.90%	0.009	0.499
Melanoma	6	2197	1.644 (1.325‐2.040)	<0.001	46.40%	0.097	0.519
MPM	2	397	1.663 (0.771‐3.589)	0.195	70.20%	0.067	0.479
Pancreatic cancer	3	465	1.731 (1.404‐2.135)	<0.001	6.60%	0.343	0.565
Prostate cancer	3	504	2.253 (1.665‐3.048)	<0.001	0%	0.463	0.903
Renal cell cancer	1	58	2.470 (0.937‐6.508)	0.067			N. E.
Unknown malignant cancer	1	193	1.567 (1.023‐2.399)	0.039			0.516
By primary tumor site							
Abdominal cancers	36	12,170	1.510 (1.366‐1.671)	<0.001	50.20%	<0.001	0.598
Head and neck cancers	10	4293	1.530 (1.265‐1.850)	<0.001	59.00%	0.009	0.704
Thoracic cancers	23	6557	1.609 (1.381‐1.876)	<0.001	42.60%	0.017	0.934
Pelvic cancers	12	3720	2.111 (1.480‐3.011)	<0.001	85.30%	<0.001	0.581
Other cancers	7	2390	1.628 (1.359‐1.950)	<0.001	35.80%	0.155	N. E.
By distant metastasis (DM)							
No DM	38	17,142	1.553 (1.361‐1.773)	<0.001	80.80%	<0.001	0.349
DM	15	3133	1.554 (1.313‐1.839)	<0.001	66.40%	<0.001	0.445
Both	30	7404	1.609 (1.450‐1.784)	<0.001	28.80%	0.073	0.617
NA	5	1451	1.780 (1.329‐2.385)	<0.001	27.40%	0.239	N. E.
By TNM stage							
<IV	28	13,268	1.513 (1.316‐1.738)	<0.001	62.00%	<0.001	0.443
IV	15	3133	1.554 (1.313‐1.839)	<0.001	66.40%	<0.001	0.611
I‐IV	37	11,457	1.693 (1.464‐1.958)	<0.001	81.00%	<0.001	0.837
NA	8	1254	1.615 (1.374‐1.898)	<0.001	0%	0.433	N. E.
By the time of blood testing							
Pre‐treatment	52	13,024	1.596 (1.418‐1.795)	<0.001	84.70%	<0.001	0.538
Pre‐operative	34	15,923	1.596 (1.418‐1.796)	<0.001	53.40%	<0.001	0.550
Post‐operative	1	34	0.800 (0.114‐5.632)	0.823			N. E.
NA	1	149	2.860 (1.377‐5.939)	0.005			0.294
By analysis method							
MV	46	19,650	1.660 (1.495‐1.843)	<0.001	57.80%	<0.001	0.244
UV	42	9480	1.524 (1.344‐1.728)	<0.001	81.60%	<0.001	N. E.
By NOS score							
High quality	52	15,300	1.596 (1.414‐1.800)	<0.001	80.70%	<0.001	N. E.
Low quality	36	13,830	1.590 (1.441‐1.754)	<0.001	50.50%	<0.001	0.680
*Disease*‐*free survival*							
Overall	41	11,514	1.488 (1.357‐1.633)	<0.001	32.00%	0.028	
By region							
Asian	35	9734	1.478 (1.329‐1.644)	<0.001	39.80%	0.009	N. E.
Non‐Asian	6	1780	1.648 (1.335‐2.036)	<0.001	0%	0.996	0.394
By sample size							
>200	21	9170	1.499 (1.304‐1.723)	<0.001	48.90%	0.006	N. E.
≤200	20	2344	1.452 (1.301‐1.621)	<0.001	3.30%	0.415	0.803
By cut‐off value							
>500	12	3679	1.538 (1.357‐1.744)	<0.001	0%	0.506	0.312
≤500	29	7835	1.453 (1.286‐1.642)	<0.001	40.00%	0.015	N. E.
By cancer							
Breast cancer	9	1550	1.465 (1.129‐1.900)	<0.001	0%	0.646	0.043
Cervical cancer	1	125	2.076 (1.063‐4.053)	0.032			0.160
Colorectal cancer	6	1857	1.738 (1.279‐2.362)	<0.001	42.70%	0.120	0.063
Endometrial cancer	2	851	1.669 (1.213‐2.295)	<0.001	0%	0.847	0.067
Esophageal cancer	3	1124	1.331 (1.077‐1.643)	0.002	0%	0.403	0.031
Extrahepatic bile duct cancer	1	121	1.939 (1.047‐3.591)	0.048			0.130
Gallbladder cancer	1	34	2.084 (0.529‐8.218)	0.294			0.280
Gastric cancer	2	460	0.805 (0.536‐1.209)	0.295	48.50%	0.163	0.006
Hepatocellular cancer	4	906	1.536 (1.116‐2.112)	0.008	65.70%	0.033	0.033
Head and neck cancer	6	3132	1.570 (1.291‐1.909)	0.026	32.70%	0.191	0.043
Lung cancer	2	735	1.474 (1.152‐1.888)	0.006	0%	0.417	0.045
Melanoma	2	227	1.530 (1.004‐2.332)	0.048	0%	0.907	0.056
Pancreatic cancer	1	144	1.471 (1.014‐2.135)	0.042			0.048
Prostate cancer	1	248	1.894 (0.998‐3.595)	0.051			0.126
By primary tumor site							
Head and neck cancers	5	3016	1.585 (1.203‐2.088)	0.001	41.60%	0.144	N. E.
Thoracic cancers	13	2867	1.418 (1.226‐1.641)	<0.001	0%	0.709	0.703
Abdominal cancers	16	3638	1.489 (1.244‐1.782)	<0.001	61.90%	0.001	0.610
Pelvic cancers	5	1766	1.623 (1.305‐2.017)	<0.001	0%	0.800	0.798
Other cancers	2	227	1.530 (1.004‐2.332)	0.048	0%	0.907	0.937
By distant metastasis (DM) status							
No DM	30	8196	1.468 (1.312‐1.642)	<0.001	41.40%	0.010	0.455
DM	1	89	1.143 (0.694‐1.883)	0.600			N. E.
Both	7	1986	1.627 (1.315‐2.013)	<0.001	0%	0.518	0.282
NA	3	1243	1.727 (1.269‐2.351)	0.001	0%	0.932	0.270
By TNM stage							
<IV stage	9	4743	1.436 (1.239‐1.664)	<0.001	42.00%	0.026	0.703
IV stage	2	163	1.279 (0.855‐1.914)	0.231	0%	0.453	N. E.
I‐IV stage	7	6019	1.524 (1.359‐1.710)	<0.001	1.30%	0.436	0.411
NA	4	589	1.931 (1.066‐3.498)	0.030	66.80%	0.029	0.555
By the time of blood testing							
Pre‐treatment	12	4092	1.498 (1.323‐1.697)	<0.001	0%	0.859	0.688
Pre‐operative	28	7388	1.500 (1.321‐1.703)	<0.001	47.60%	0.003	0.692
Post‐operative	1	34	2.084 (0.529‐8.218)	0.294			N. E.
By analysis method							
MV	16	6277	1.420 (1.248‐1.616)	<0.001	44.40%	0.029	0.274
UV	25	5237	1.566 (1.375‐1.784)	<0.001	18.10%	0.209	N. E.
By NOS score							
High quality	29	7126	1.438 (1.293‐1.599)	<0.001	32.50%	0.048	0.236
Low quality	12	4388	1.665 (1.371‐2.021)	<0.001	31.10%	0.142	N. E.
*Progressive*‐*free survival*							
Overall	23	5126	1.533 (1.342‐1.751)	<0.001	45.50%	0.010	
By region							
Asian	16	3852	1.672 (1.380‐2.026)	<0.001	48.60%	0.015	0.126
Non‐Asian	7	1274	1.290 (1.145‐1.454)	<0.001	0%	0.531	N. E.
By sample size							
>200	10	3769	1.655 (1.348‐2.031)	<0.001	50.50%	0.033	0.409
≤200	13	1357	1.442 (1.209‐1.720)	<0.001	38.30%	0.078	N. E.
By cut‐off value							
>500	13	2359	1.439 (1.184‐1.749)	<0.001	54.60%	0.009	0.967
≤500	9	2472	1.700 (1.442‐2.004)	<0.001	1.30%	0.423	N. E.
NA	1	295	1.380 (1.062‐1.793)	0.016			0.152
By cancer type							
Cervical cancer	5	1981	2.338 (1.091‐5.011)	0.029	81.90%	<0.001	0.460
Colorectal cancer	4	1094	1.487 (1.259‐1.756)	<0.001	0%	0.667	0.610
Endometrial cancer	1	310	1.397 (0.938‐2.080)	0.100			0.471
Esophageal cancer	1	126	1.900 (1.110‐3.251)	0.019			0.761
Head and neck cancer	1	75	2.923 (1.214‐7.036)	0.017			0.259
Lung cancer	6	921	1.381 (1.161‐1.642)	<0.001	0%	0.445	0.805
Pancreatic cancer	1	57	1.188 (0.419‐3.369)	0.746			0.963
Prostate cancer	3	504	1.791 (1.310‐2.449)	<0.001	33.20%	0.224	0.439
Renal cell cancer	1	58	1.140 (0.521‐2.497)	0.743			N. E.
By primary tumor site							
Head and neck cancers	1	75	2.923 (1.214‐7.036)	0.017			0.176
Thoracic cancers	6	921	1.318 (1.161‐1.642)	<0.001	0%	0.445	N. E.
Abdominal cancers	7	1335	1.494 (1.281‐1.743)	<0.001	61.90%	0.811	0.501
Pelvic cancers	9	2795	1.842 (1.339‐2.533)	<0.001	72.80%	<0.001	0.248
By distant metastatic status							
No DM	7	2025	1.876 (1.173‐3.000)	0.009	72.20%	0.001	N. E.
DM	5	855	1.640 (1.361‐1.975)	<0.001	0%	0.423	0.736
Both	11	2246	1.436 (1.253‐1.646)	<0.001	12.20%	0.328	0.694
By TNM stage							
<IV	3	506	2.892 (0.945‐8.850)	0.063	75.80%	0.016	N. E.
IV	5	855	1.640 (1.361‐1.975)	<0.001	0%	0.423	0.250
I‐IV	13	3482	1.378 (1.187‐1.600)	<0.001	33.90%	0.111	0.077
NA	2	283	1.608 (1.202‐2.152)	0.001	0%	0.469	0.265
By the time of blood testing							
Pre‐treatment	20	4367	1.469 (1.302‐1.658)	<0.001	30.90%	0.094	0.272
Pre‐operative	3	759	2.717 (1.093‐6.757)	0.032	81.30%	0.005	N. E.
By analysis method							
MV	13	3334	1.762 (1.478‐2.102)	<0.001	35.90%	0.095	0.011
UV	10	1792	1.276 (1.112‐1.465)	0.001	14.00%	0.314	N. E.
By NOS score							
High quality	14	1992	1.396 (1.221‐1.596)	<0.001	23.70%	0.198	0.172
Low quality	9	3134	1.805 (1.377‐2.366)	<0.001	56.50%	0.019	N. E.
*Cancer*‐*specific survival*							
<0.001	57.00%	0.013	1.585 (1.253‐2.006)				
By region							
Asian	7	1780	1.593 (1.179‐2.152)	0.002	64.90%	0.009	0.952
Non‐Asian	3	535	1.621 (1.055‐2.491)	0.028	41.30%	0.182	N. E.
By sample size							
>200	5	1590	1.780 (1.197‐2.648)	0.004	62.80%	0.029	N. E.
≤200	5	725	1.454 (1.074‐1.970)	0.016	52.80%	0.076	0.549
By cut‐off value							
>500	5	1080	1.484 (1.195‐1.843)	<0.001	9.30%	0.353	0.754
≤500	5	1235	1.772 (1.106‐2.839)	0.017	75.20%	0.003	N. E.
By cancer type							
Colorectal cancer	5	1080	1.524 (1.209‐1.921)	<0.001	0%	0.482	0.651
Hepatocellular cancer	1	198	1.070 (0.835‐1.371)	0.593			0.369
Head and neck cancer	2	399	3.339 (0.742‐15.026)	0.116	83.50%	0.014	0.821
Lung cancer	1	348	1.282 (0.897‐1.832)	0.173			0.496
Prostate cancer	1	290	2.240 (1.282‐3.914)	<0.001			N. E.
By primary tumor site							
Head and neck cancers	2	399	3.339 (0.742‐15.026)	0.116	83.50%	0.014	0.313
Thoracic cancers	1	348	1.282 (0.897‐1.832)	0.173			N. E.
Abdominal cancers	6	1278	1.373 (1.087‐1.734)	0.008	34.60%	0.177	0.815
Pelvic cancers	1	290	2.240 (1.282‐3.914)	0.005			0.451
By metastatic status							
No DM	2	497	1.975 (0.699‐5.579)	0.199	74.30%	0.049	0.322
DM	4	680	1.508 (1.208‐1.881)	0.019	0%	0.916	0.176
Both	3	940	2.579 (1.168‐5.695)	<0.001	68.90%	0.040	0.043
NA	1	198	1.070 (0.835‐1.371)	0.593			N. E.
By TNM stage							
<IV	1	149	3.780 (1.372‐10.415)	0.010			N. E.
IV	4	680	1.508 (1.208‐1.881)	<0.001	0%	0.916	0.143
I‐IV	3	940	2.579 (1.168‐5.695)	0.019	68.90%	0.040	0.648
NA	2	546	1.135 (0.926‐1.391)	0.224	0%	0.415	0.071
By the time of blood testing							
Pre‐treatment	3	689	2.577 (1.331‐4.989)	0.005	67.20%	0.048	N. E.
Pre‐operative	5	1393	1.253 (1.066‐1.473)	0.006	0%	0.526	0.111
NA	2	233	2.038 (0.738‐5.626)	0.169	69.10%	0.072	0.570
By analysis method							
MV	6	1583	1.600 (1.126‐2.272)	0.009	69.10%	0.006	0.915
UV	4	732	1.595 (1.210‐2.101)	0.001	14.90%	0.317	N. E.
By NOS score							
High quality	7	1634	1.577 (1.196‐2.078)	0.001	65.10%	0.009	N. E.
Low quality	3	681	1.680 (0.978‐2.884)	0.060	41.00%	0.183	0.751

NOS: Newcastle‐Ottawa Quality Assessment Scale; OS: overall survival; DFS: disease‐free survival; PFS: progression‐free survival; CSS: cancer‐specific survival; HR: hazard ratio; MPM: malignant pleural mesothelioma; DM: distant metastasis; NA: not available information; MV: multivariate analysis; UV: univariate analysis; 95%CI: confidence interval; P: *p* value of pooled HR; *I* ^2^: value of Higgins I‐squared statistics; Ph: p value of Heterogeneity test; N. E.: not estimation.

^a^ Numbers of studies included in the meta‐analysis.

^b^ Number of included patients.

### 3.3 Disease‐free survival

**FIGURE 4:**
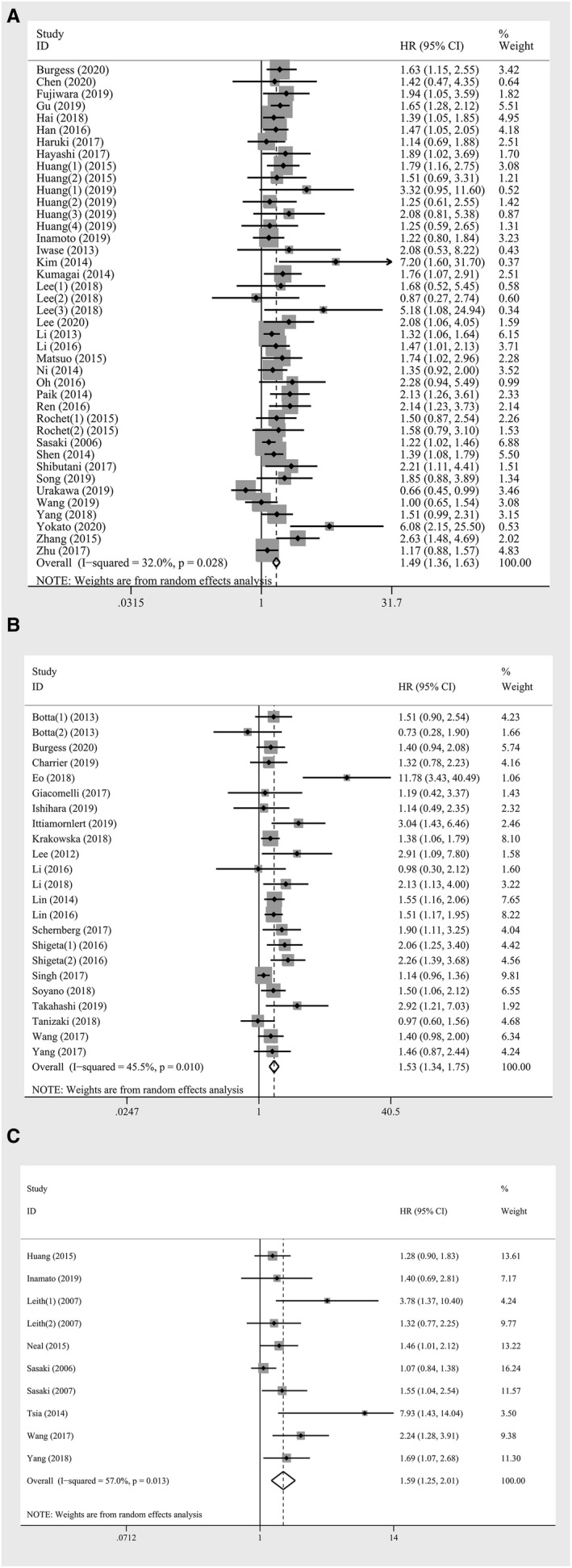
Forest plot of meta‐analysis of the prognostic role of absolute monocyte count for (A) disease‐free survival (B) progression‐free survival (C) cancer‐specific survival in solid tumors

There were 41 studies, comprising 11,514 patients, reporting HRs for DFS. Overall, high AMC was significantly associated with worse DFS *(HR*:*1*.*488*; *95% CI*: *1*.*357*‐*1*.*633*; *p* < *0*.*001)* (Figure 4). Low heterogeneity was shown between these studies *(I^2^* = *32*.*00%*; *p* = *0*.*028)*. The analyses demonstrated that elevated AMC was positively related to pooled HR for DFS when stratified by region, sample size, cut‐off value, analysis method, and study quality. In subgroup analyses according to cancer type, studies on breast cancer, endometrial cancer, esophageal cancer, lung cancer, and melanoma also demonstrated the negative effect of high AMC on outcomes with consistency. In addition, we found poor DFS in studies with IV stage cancers, but the estimate was insignificant *(HR*:*1*.*279*; *95% CI*: *0*.*855*‐*1*.*914*; *p* = *0*.*231)* (Table 2).

### 3.4 Progressive‐free survival

Data on the association between AMC and PFS were derived from 23 studies involving 5,126 patients. Overall higher AMC associated with worse prognosis *(HR*: *1*.*533*; *95% CI*: *1*.*342*‐*1*.*751*; *p* < *0*.*001)*, with moderate heterogeneity *(I^2^* = *45*.*50%*; *p* = *0*.*010)* (Table 2, Figure 4). Exploratory subgroup analyses were performed according to region, cancer type and TNM stages, and a prognostic role of AMC was observed in non‐Asian patients *(HR*: *1*.*274*, *95% CI*: *1*.*145*‐*1*.*454*; *p* < *0*.*001)*, patients with colorectal cancer *(HR*: *1*.*487*; *95% CI*: *1*.*259*‐*1*.*756*; *p* < *0*.*001)* and lung cancer *(HR*: *1*.*381*; *95% CI*: *1*.*161*‐*1*.*642*; *p* < *0*.*001)*, patients with stage IV cancer *(HR*: *1*.*640*; *95% CI*: *1*.*361*‐*1*.*975*; *p* < *0*.*001)* with no heterogeneity. The adverse effect of higher AMC was also seen when stratified by sample size, cut‐off value, distant metastatic status, the time of blood testing, analysis method, and study quality (Table 2).

### 3.5 Cancer‐specific survival

Ten studies involving 2,315 patients reported suitable data for CSS analysis. Overall, an increase in the monocyte showed associations with worse CSS *(HR*: *1*.*585*; *95% CI*: *1*.*253*‐*2*.*006*; *p* < *0*.*001)* (Table 2; Figure 4). A relatively high heterogeneity was observed across the studies *(I^2^* = *57*.*00%*; *p* = *0*.*013)*. When stratified by cancer type, distant metastatic status, and the time of blood testing, elevated AMC was associated with worse outcome in patients with colorectal cancer *(HR*: *1*.*523*; *95% CI*: *1*.*209*‐*1*.*921*; *p* < *0*.*001)*, distant metastatic cancer *(HR*: *1*.*508*; *95% CI*: *1*.*208*‐*1*.*881*; *p* < *0*.*001)* and studies with pre‐operative AMC *(HR*: *1*.*253*; *95% CI*: *1*.*066*‐*1*.*473*; *p* = *0*.*006)* without heterogeneity. The subgroup analyses also revealed that elevated AMC might be a potential biomarker in non‐Asian patients, studies with high cut‐off value, and studies with univariate analysis or low quality (Table 2).

### 3.6 Relationships of AMC and clinical features

Association between monocyte count and clinicopathological parameters were evaluated among 28 studies. There was a significantly positive associations between elevated AMC and advanced T stage *(OR*:*1*.*298*; *95% CI*:*1*.*035*‐*1*.*629*; *p* = *0*.*024)*, microvascular invasion *(OR*:*1*.*896*; *95% CI*:*1*.*240*‐*2*.*900*; *p* = *0*.*003)*, macrovascular invasion *(OR*:*4*.*713*; *95% CI*: *1*.*293*‐*17*.*177*; *p* = *0*.*019)* and larger tumor length *(OR*:*1*.*783*; *95% CI*:*1*.*378*‐*2*.*308*; *p* < *0*.*001)*. The pooled analysis revealed that the AMC of male with solid tumors was more likely higher *(OR*:*2*.*147*; *95% CI*: *1*.*650*‐*2*.*795*, *p* < *0*.*001)*. (Table 3, Figure S1) We also evaluated that smoking was positively associated with higher AMC *(OR*:*1*.*684*, *95% CI*: *1*.*104*‐*2*.*570*, *p* = *0*.*016)*. Patients with elevated pre‐operative monocyte has lower albumin *(SMD*: *0*.*264*; *95% CI*: *0*.*084*‐*0*.*444*; *p* = *0*.*004)*, while higher platelet *(SMD*:*0*.*455*; *95% CI*:*0*.*166*‐*0*.*743*; *p* = *0*.*002)* (Table 3).

**TABLE 3:** Meta‐analysis of the association between elevated AMC and clinicopathological features of cancers

Variables	Studies	Patients	Pooled OR	95% CI	*p* value*	Heterogeneity *I* ^2^	Ph value**
Dichotomous variables							
Gender (male vs. female)	19	4340	2.147	1.650‐2.795	<0.001	65.50%	<0.001
APF (high vs. low)	5	1102	1.265	0.965‐1.659	0.089	4.60%	0.381
Distant metastasis	5	1405	1.179	0.921‐1.510	0.192	0%	0.718
T stage (T2+ vs. <T2)	9	4222	1.298	1.035‐1.629	0.024	18.70%	0.277
N stage (N1+ vs. N0)	9	4324	1.080	0.910‐1.281	0.379	20.00%	0.265
TMN stage (III+IV vs. I+II)	9	2503	1.224	0.950‐1.576	0.118	47.50%	0.055
Microvascular invasion (yes vs. no)	3	555	1.896	1.240‐2.900	0.003	0%	0.620
Macrovascular invasion (yes vs. no)	2	452	4.713	1.293‐17.177	0.019	83.90%	0.013
Vascular invasion (yes vs. no)	3	740	1.580	0.975‐2.561	0.063	0%	0.700
Lymphatic permeation (yes vs. no)	2	520	0.943	0.537‐1.655	0.837	18.80%	0.267
Tumor length (high vs. low)	8	1520	1.783	1.378‐2.308	<0.001	8.20%	0.367
Differentiation (well vs. poor/moderate)	10	2625	0.968	0.784‐1.196	0.762	9.40%	0.356
Adjuvant therapy^a^	6	1422	0.900	0.715‐1.133	0.370	0%	0.670
Adjuvant therapy^b^	3	883	0.905	0.675‐1.214	0.505	0%	0.651
Smoking (yes vs. no)	6	1858	1.684	1.104‐2.570	0.016	72.80%	0.002
Continuous variables							
Age	11	3072	0.019	−0.077‐0.116	0.693	39.10%	0.088
Hemoglobin	4	1025	0.004	−0.119‐0.128	0.946	0%	0.707
Platelet	4	1008	0.455	0.166‐0.743	0.002	72.00%	0.013
Albumin (low vs. high)	3	790	0.264	0.084‐0.444	0.004	0%	0.471
Hematocrit	3	807	−0.014	−0.156‐0.124	0.841	0%	0.690

OR: odd ratio; SMD: standardized mean difference; CI: confidence interval; *I* ^2^: the value of I‐squared statistics; vs.: versus.

^a^ Studies with adjuvant therapy versus studies without adjuvant therapy.

^b^ Studies with radiotherapy versus studies with concurrent chemoradiotherapy.

* *p* value of pooled HR.

** *p* value of Heterogeneity test.

*** *p* value of SMD.

### 3.7 Publication bias and sensitivity analysis

For OS, the funnel plot was visibly asymmetrical, indicating the presence of publication bias (Figure S2). In accord with the plot, the results of Begg's test *(p* = *0*.*028)* and Egger's test *(p* < *0*.*001)* further confirmed that the asymmetry was mainly attributed to the publication bias. The “trim‐and‐fill” analysis was performed and no significant change in our results was found, further suggesting the stability of the meta‐analysis *(HR*: *1*.*461*; *95% CI*: *1*.*343*‐*1*.*588*; *p* < *0*.*001)* (Supplementary Figure 3). For DFS, the results of Begg's test *(p* = *0*.*003)* and Egger's test *(p* = *0*.*001)* showed publication bias. The adjusted random effect HR of 1.363 *(95% CI*: *1*.*234*‐*1*.*506*, *p* < *0*.*001)* was obtained using the “trim‐and‐fill” analysis, which was consistent with the primary analysis. For PFS, there were no evidence of asymmetry, and no trimming was performed after applying the “trim‐and‐fill” analysis. For CSS, the results of Begg's test *(p* = *0*.*007)* and Egger's test *(p* = *0*.*001)* indicated publication bias, and there was no trimming performed after applying the “trim‐and‐fill” analysis.

In addition, sensitivity analysis was carried out and the results showed that the pooled HRs were not significantly affected by omitting an individual study.

## 4 DISCUSSION

To the best of our knowledge, we performed a comprehensive meta‐analysis for the first time to assess the prognostic value of AMC in various solid tumors. The systematic review and meta‐analysis involving data on 32,229 participants from 104 studies provided robust evidence that elevated the level of monocyte count might be an independent prognostic factor for poor OS, DFS, PFS, and CSS in non‐hematologic tumors. Subgroup analyses focused on clinical outcomes were conducted and further proved the predictor role of elevated AMC on long‐term cancer outcomes. In addition, we found a tendency that an elevated AMC was significantly associated with some clinicopathological characteristics including gender, T stage, vascular invasion, tumor length, and smoking, as well as higher platelet counts and lower albumin.

Since Virchow described the role of inflammation in 1863, inflammatory response has gone beyond the marker of infection. It has been hypothesized that cancer arises from the background of inflammation, which has been supported by a multitude of evidence in the last decade.^10^, ^11^, ^12^ So far, it has been widely accepted that the inflammatory components, which constitute a major part of the TME, may be triggered by the conditions that predisposes to cancer or by genetic events.^6^ What is more, as a binary “anti‐tumor” or “pro‐tumor” environment, the dominant function of TME is determined by the cross‐regulation of immune cells and non‐immune cells by perceiving signaling molecules to induce the proliferative activity of the tumor, metastasis, cell migration, and immune response against therapy.^7^, ^11^ Recent research efforts have shed light on the prognostic significance of immune cells in various cancers.^21^, ^22^, ^23^, ^24^, ^25^, ^26^

Of these immune cells, monocytes are a subset of circulating blood cell originated from myeloid progenitors in the bone marrow and subsequently can be attracted to peripheral tissues via bloodstream.^120^ Circulating monocytes perform versatile functions both in antimicrobial defense and chronic inflammation. In TME, peripheral monocytes constantly enter the tumor sites and inhibit the tumor‐related immune defense function by expressing inhibitory molecules and/or releasing soluble inhibitory factors via tumor‐derived signals.^121^, ^122^ As monocyte measurement is easily standardized and available in blood routine examination, monocyte could be a potentially helpful and convenient serum biomarker in clinical practice even be added as a new item in the immunoscore system.^14^ The qualitative and quantitative changes of the monocyte count in tumor have been attracting research attention. In accordance with our finding, recent meta‐analyses also pooled the data of individual tumor types and demonstrated that elevated peripheral monocyte appeared to be synonymous with an increased risk of mortality in the context of several malignancies.^123^, ^124^ However, the effect of elevated AMC on outcomes has not been synthetically and comprehensively analyzed in solid tumors and the variation of the effects according to tumor type has not been explained. In addition, the direct evidence for some cancers, such as melanoma and malignant pleural mesothelioma, was sparse due to limited studies and small sample sizes. Therefore, we conducted the present meta‐analysis to sort through currently available data and concluded that elevated AMC was associated with poor clinical outcomes in non‐hematologic tumors.

The exact underlying mechanisms of the association between elevated AMC and unfavorable outcomes have not been fully clarified and might be multifactorial. It is recognized that monocytes originate from mononuclear myeloid cells in bone marrow, which come into play in response to pathogenic stimuli such as cancer by myelopoiesis which largely manifest in the expansion of monocytes and neutrophils.^125^, ^126^ In the study, our results aggregated previous studies and revealed the positive associations between elevated AMC and local invasion of tumor cells and tumor length. Besides, TAMs plays a vital role at the crossroads of inflammation and cancer.^6^, ^7^ Monocytes travel to peripheral tissue and move directionally to the tumor sites owning to tumor‐derived signals, subsequently differentiate to TAMs.^12^, ^18^ Anti‐tumor M1 macrophages are characterized by the induction of lipopolysaccharide (LPS) and IFN‐γ, and are able to withstand intracellular pathogens and cancer cells.^15^, ^16^ In contrast, polarized pro‐tumor M2 macrophages are a source and target of a distinct pattern of cytokines, chemokines, and growth factors generally exerting tumor‐promoting and immune escape effects, and impairs anti‐cancer therapies.^127^, ^128^ Therefore, the understanding of the balance between M1 and M2 polarization provides a theoretical foundation for better rational manipulation of monocytes differentiation and macrophage polarization switching in TME.^128^ Monocyte subpopulations have different functions in TME.^129^ Previous study described that inflammatory monocytes could be predominantly divided into classical inflammatory monocytes (CCR2^high^Ly6C^++^CD43^+^ in mice, homologous to CCR2^high^CD14^++^CD16^−^ in human), intermediate monocytes (Ly6C^++^CD43^++^ in mice, homologous to CD14^++^CD16^+^ in human) and nonclassical patrolling monocytes (CX_3_CR1^high^Ly6C^+^CD43^++^ in mice, homologous to CX_3_CR^high^CD14^+^CD16^++^ in human). (The ^+^ denotes an expression level that is 10‐fold above the isotype control and ^++^ is 100‐fold above the isotype control).^129^, ^130^ Prat et al. found a significant increase in intermediate monocyte subpopulations that performed protumor function through the proangiogenic capacities in ovarian cancer.^131^ The study also explored the correlation between intermediate monocytes and protumor immunosuppressive microenvironment in ascites.^131^ It was hypothesized that the circulating monocytes might be modulated by secreted factors produced by stromal cells and tumor cells in TME, such as IL‐10 and CCL2.^131^

In addition, the monocyte chemotactic factor monocyte chemoattractant protein‐1 /C‐C chemokine ligand 2 (MCP‐1/CCL2) secreted by tumor cells is a chemokine with potent monocyte chemotactic activity via binding to CCR2 (the receptor for chemokine CCL2), has been shown to directly or indirectly enhance immunosuppression and metastasis by vascular endothelial growth factor (VEGF) secretion and other tumor‐secreted factors like IFNγ in murine cancer models.^132^, ^133^, ^134^ Yoshimura T. expatiated on the correlation between MCP‐1 production and TAMs and indicated that MCP‐1 production regulated the vicious cycle between host cells and tumor cells thus promoting cancer progression.^134^ Additionally, recent studies reported the NK cell‐monocyte interactions enhanced NK cell antitumor activity in cancer prognosis and the response to monoclonal antibody therapy.^135^, ^136^ It has been reported that tumor‐infiltrating monocytes/macrophages induced NK cell dysfunction via TGFβ1, thus impairing the expression of IFNα, TNFγ, and Ki‐67 in tumor progression in gastric cancer.^136^ Kubo et al. also demonstrated that patrolling monocytes contributing to the prevention of primary tumor by producing IL‐15, which is the key mediator to activate the anti‐metastatic role of NK cells.^137^ Moreover, myeloid‐derived suppressor cells (MDSCs) generated from myeloid cells in TME and were similar but functionally distinct from monocytes and neutrophils.^125^ Chae et al. demonstrated that MDSC could also arose directly from monocytes and displayed the immune suppressive activity in tumor progression in the murine model.^138^ However, cells with MDSC features could be easily mistaken for monocytes in some studies.^138^ Otherwise, Kuang et al. found monocytes activated by tumors strongly express programmed cell death ligand 1 (PD‐L1) and effectively suppressed tumor‐specific T cell immunity in HCC in vivo.^139^ Taube et al. also demonstrated some factors such as IL‐10 and IL‐32γ induced PD‐L1 expression on monocytes in melanoma.^140^ Therefore, the PD‐L1 expression on the monocytes could be a novel mechanism explaining the association between monocyte and cancer. All the proposed mechanisms about monocyte might inspire potential molecular targets for personalized treatment strategy of solid tumors.

It has been reported that a therapeutic regimen such as chemotherapy could modulate the pro‐tumor and anti‐tumor ability of monocytes/macrophages lineages.^141^ Different oncotherapy regimens have been supposed to be associated with various changes in the biology and function of these cells.^141^ In the subgroup analyses, nevertheless, studies on the prognostic value of AMC in patients with different treatments have not been evaluated due to the variety and complexity of treatment programs. In addition, our meta‐analysis has demonstrated that elevated AMC was associated with poor prognosis. However, a precise cut‐off value in clinical practice is a matter of broad discussion. At present, ROC curves were widely used in the threshold selection in diagnostic or screening tests considering the optimization of false‐positive and false‐negative interpretation. For this reason, most of the included studies chose ROC curves to define their threshold cut‐off values. In the paper, we split studies with a cut‐off >500 or ≤500, both studies with high cut‐off and with low cut‐off were associated with an increased risk of worse outcomes.

Our study possesses several strengths. Distinctively, we focused on all non‐hematological tumors and provided comprehensive evidence for the prognostic value of AMC, which might be a weighted one involved in the immunoscore system. Moreover, because of the diversity of cancer types and studies, the research into subgroup analysis were lucubrated. It is the first time that pre‐treatment AMC, pre‐operative AMC, and post‐operative AMC are compared. Based on subgroup analyses stratified by cut‐off values of monocytes, higher cut‐off values for OS and DFS seem to be more discriminative effective on prognosis, while inversely in PFS and CSS. Additionally, we studied the relationships between AMC and gender, T stage, tumor length, microvascular invasion, smoking, and other clinic parameters, which provide potential implications in clinical practice in the future.

Nevertheless, there were some limitations to the present study. A key limit was that cut‐off levels of AMC were set based on ROC, median value, previous studies or other methods, making the routine application less practical. Although the cut‐off >500/mm^3^ may enable us to better identify the poor outcomes in the present study, the optimal AMC cut‐off awaits standardization. Besides, as Walker SP said, “the unpredictability of the diseases undermined the ability to plan ahead.”^142^ In practice, the dynamic change in prognostic indicators might be more valuable for one patient with solid tumor. Future studies on changes in cancer biomarkers and cut‐off values defining the contributions of each cancer type are required. Additionally, we failed to find the sources of the heterogeneity of overall survival analyses with subgroup analyses and meta‐regression analyses. Due to the variations of study quality and sample size among included studies, the statistical methods might be refined and a weighted mean might be computed. As mentioned before, publication bias existed in OS, DFS, and CSS analyses, and we were unable to extract the unreported data in some studies. Moreover, numerous confounding factors influence the post‐operative AMC, such as surgical stress, bleeding, sepsis, even wound healing. Therefore, the prognostic value of post‐operative AMC is relatively rarely reported. In addition, the relationships between monocyte and the features of tumor patients were not well defined in our study because of the lack of original data, hence the results may be less suitable in clinical practice.

## 5 CONCLUSIONS

In conclusion, our comprehensive meta‐analysis strongly supported that elevated AMC was remarkably associated with poor prognosis of patients with solid cancer. Monocyte, the relatively accessible low‐cost cancer biomarker, could have widespread clinical implications for surgical management, treatment strategy, and prognosis assessment. Further multicenter studies in a randomized and prospective manner with optimal AMC cut‐offs were warranted to refine our results and to advance the clinical applications of the monocyte counts in the future. Finally, given the different functions of monocyte subsets in TME, the monitoring of blood monocyte subpopulations could be further explored and applied to follow‐up treatment response.

## 6 DATA AVAILABLE STATEMENT

This study reports a systematic review for which all data are already available within the public realm in the form of scientific publications, references for which are provided.

## CONFLICTS OF INTEREST

The authors have declared no conflict of interest.

## AUTHOR CONTRIBUTIONS

Shu Wen, Ying Hu, and Liangzhi Xu conceived and designed the experiments; Litao Huang, Jin Peng, and Nan Chen collected the data; Meina Yang and Xiaoyang Shen analyzed the data; Shu Wen and Nan Chen contributed the materials/analysis tools and wrote the manuscript. Ying Hu and Song Yang revised the manuscript. All authors reviewed and approved the manuscript prior to submission.

## Supporting information

Fig S1

Fig S2

Fig S3
