# Supplementary figures and images for: Elevated peripheral absolute monocyte count related to clinicopathological features and poor prognosis in solid tumors: Systematic review, meta‐analysis, and meta‐regression
Source: Cancer Med. 2021 Feb 16;10(5):1690–714. doi: 10.1002/cam4.3773 (PMC7940224; doi:10.1002/cam4.3773)

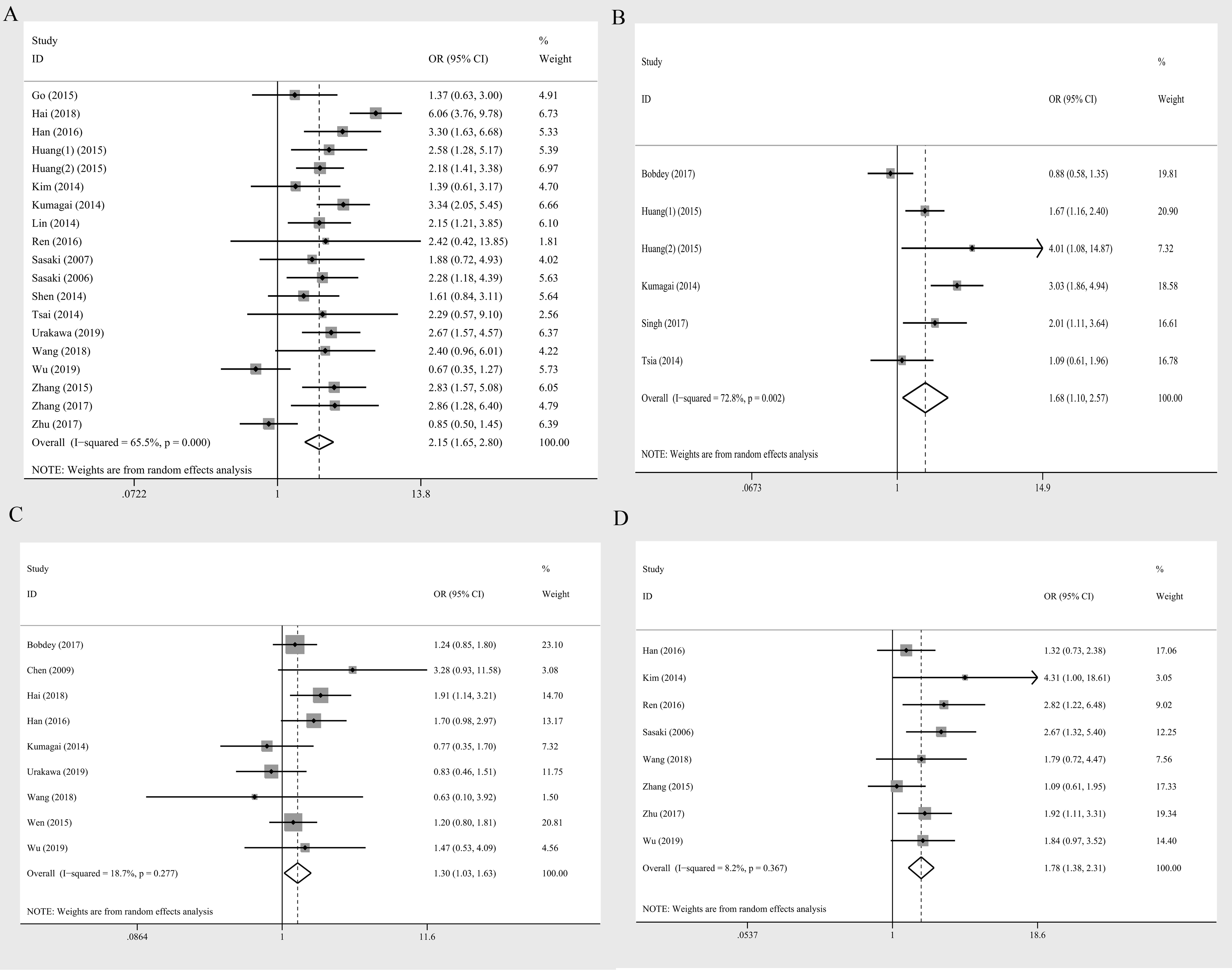

Supplement: Supplementary file 1 — Fig S1 [file CAM4-10-1690-s002.tif]

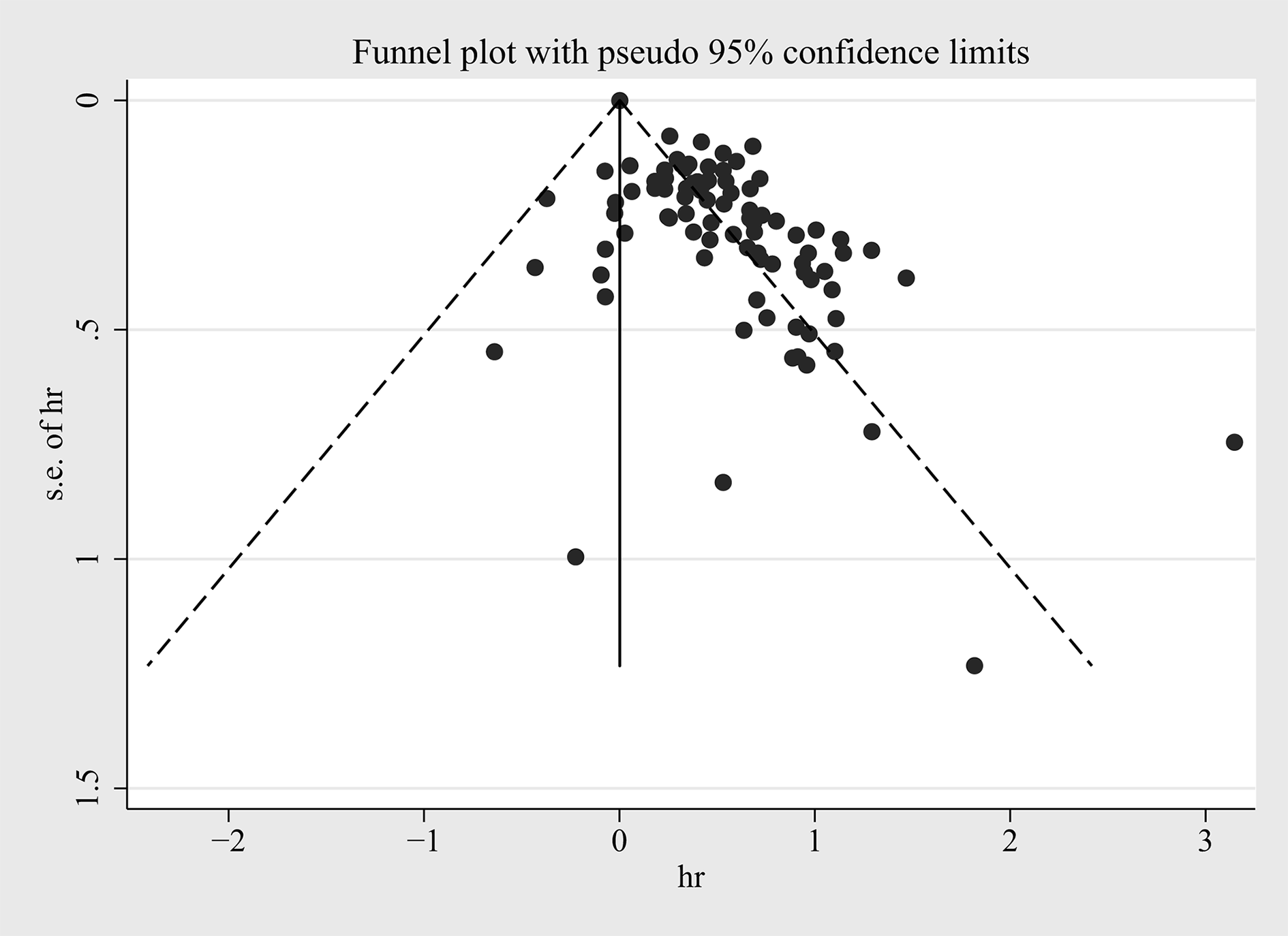

Supplement: Supplementary file 2 — Fig S2 [file CAM4-10-1690-s001.tif]

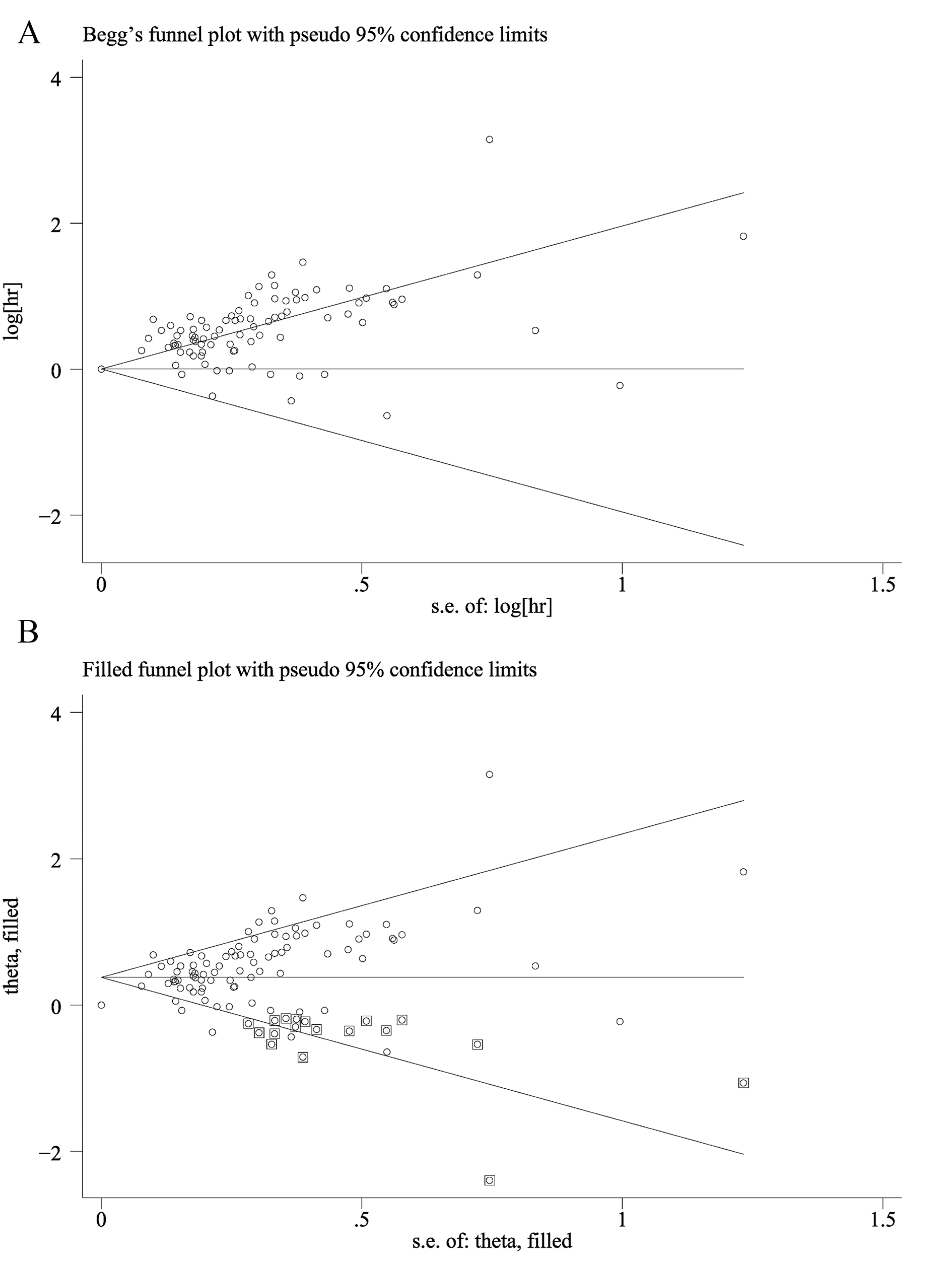

Supplement: Supplementary file 3 — Fig S3 [file CAM4-10-1690-s003.tif]
